# Predicting the outcome of specialist rehabilitation after non-traumatic subarachnoid haemorrhage: a multicentre cohort analysis from the UK Rehabilitation Outcomes Collaborative (UKROC) database

**DOI:** 10.1136/bmjno-2025-001231

**Published:** 2026-04-09

**Authors:** Alasdair Sandland, Sachin Watve, Kathleen Baster, Lynne Turner-Stokes, Krishnan Padmakumari Sivaraman Nair

**Affiliations:** 1 Medical School, The University of Sheffield, Sheffield, UK; 2 Neurosciences, Sheffield Teaching Hospitals NHS Foundation Trust, Sheffield, UK; 3 Central Statistical Support Unit, The University of Sheffield, Sheffield, UK; 4 Department of Palliative Care Policy and Rehabilitation, King’s College London, London, UK; 5 Northwick Park Hospital, London, UK; 6 Neurology, Sheffield Teaching Hospitals NHS Foundation Trust, Sheffield, UK; 7 Neurosciences, The University of Sheffield, Sheffield, UK

**Keywords:** SUBARACHNOID HAEMORRHAGE, REHABILITATION, ACQUIRED BRAIN INJURY

## Abstract

**Background and methods:**

Non-traumatic subarachnoid haemorrhage (ntSAH) is associated with high mortality and disability. This cohort analysis aims to assess the impact of inpatient rehabilitation after ntSAH using the UK Rehabilitation Outcomes Collaborative database 2012–2023. Patients who gained a Minimal Clinically Important Difference (MCID) of 26.2 on functional independence (UK Functional Independence Measure+Functional Assessment Measure (UK FIM+FAM)) were compared with those who did not, using binary logistic regression to assess for predictors of positive outcomes.

**Results:**

Among 2928 patients with ntSAH (1209 men and 1719 women, mean age 58 years (SD 13.2)), 1287 achieved MCID. Following inpatient rehabilitation, the mean total UK FIM+FAM improved by 42.3 points (95% CI 41.0 to 43.6, p=0.001), care needs reduced by a mean of 26.6 hours/week (95% CI 25.2 to 28.1, p=0.001) and care costs by a mean of £1998/week (95% CI 1872 to 2124, p=0.001). Admission to rehabilitation within 30 days of referral was associated with achieving MCID (1.638–2.673, p<0.001). Achieving MCID in either cognitive (1.730–3.180, p<0.001) or motor (1.527–4.893, p<0.001) subsections was associated with significantly increased odds of home discharge.

**Conclusions:**

Rehabilitation improves functional outcomes following ntSAH. Predictors of achieving MCID include ethnicity, time from referral to admission and admission Patient Categorisation Tool scores. Predictors of home discharge include length of stay, dependency on admission and achieving MCID in Motor and/or Cognitive FIM domains.

**WHAT IS ALREADY KNOWN ON THIS TOPIC**

- Inpatient rehabilitation is an effective and cost efficient method of improving the functional status of patients with acquired brain injuries, such as traumatic brain injury.

**WHAT THIS STUDY ADDS**

- Post-acute inpatient rehabilitation is effective and cost efficient in non-traumatic subarachnoid haemorrhage (ntSAH) across all age groups. White British ethnicity and early admission to a specialised rehabilitation unit are some of the key predictors of good outcomes. Gains in cognitive and motor activities improve the odds of discharge to home.

**HOW THIS STUDY MIGHT AFFECT RESEARCH, PRACTICE OR POLICY**

- This study provides evidence to support early inpatient rehabilitation for all patients with ntSAH, irrespective of age and dependency. Further research is warranted to understand the impact of ethnicity on rehabilitation outcomes.

## Introduction

 The incidence of spontaneous non-traumatic subarachnoid haemorrhage (ntSAH) is between 6 and 9 per 100 000 per year.^1 2^ With improvements in critical care, the mortality due to ntSAH has declined, and the number of survivors living with disabilities has increased.^3^ Up to one-third of survivors of ntSAH experience significant disabilities and require assistance for activities of daily living and mobility.^4 5^ Independent predictors of poor clinical outcomes following ntSAH include age, severity of initial clinical presentation, preoperative rebleeding, delayed cerebral ischaemia and hydrocephalus.^6^,^8^ Survivors of ntSAH often require inpatient rehabilitation services following acute management. The cost of inpatient rehabilitation following acute treatment of ntSAH accounts for about 15% of the total cost of the initial episode of care.^9^ There are no large multicentre studies on outcomes of people undergoing rehabilitation following ntSAH.

In England, inpatient rehabilitation services are divided into Level 3 units (local general), Level 2 units (district specialist units managing a more complex caseload) and Level 1 units (tertiary regional units taking a selected population of patients with highly complex rehabilitation needs). The UK Rehabilitation Outcomes Collaborative (UKROC) clinical registry is a national database which collates patient-level data on needs, inputs, outcomes and costs from all 75 Level 1 and Level 2 specialist inpatient rehabilitation services in England. Evidence from analyses of UKROC data demonstrates that patients who underwent rehabilitation following acute neurological problems such as traumatic brain injury, stroke and spinal cord injuries had improved functional independence, and rehabilitation reduced the cost of care.^10^,^12^

In this study of the UKROC dataset, we examine the outcomes of survivors of ntSAH undergoing specialist neurological rehabilitation and the predictors of a favourable outcome. We also explore the factors associated with discharge to home.

## Methods

### Study design

This is a retrospective observational cohort analysis of prospectively collected data from UKROC.

### Data source and principal measurements

This analysis uses de-identified patient data collected as a mandatory requirement of standard clinical practice of specialist rehabilitation services in England and collated within the UKROC database. In addition to basic demographic data, the UKROC database systematically records information on rehabilitation needs, inputs and outcomes on admission to and discharge from the rehabilitation service using a number of validated measures.^13^ The measures used in this analysis were the UK Functional Independence Measure+Functional Assessment Measure (UK FIM+FAM), the Rehabilitation Complexity Scale V.13 (RCS-E), the Patient Categorisation Tool (PCAT), the Northwick Park Dependency Scale (NPDS) and the Northwick Park Care Needs Assessment (NPCNA).

The UK FIM+FAM has 30 items, which combine the 18-item FIM V.4 with 12 additional FAM items primarily addressing cognitive and psychosocial function.^14^ Each item is scored from 1 (total dependence) to 7 (complete independence), giving a total score between 30 and 210, with higher scores indicating greater levels of independence. The sum of the scores from the 16 motor items (comprising self-care, sphincter control and locomotion) forms the FIM+FAM motor subscore (16–112), and the 14 cognitive items (comprising communication, psychosocial and cognitive function) form the FIM+FAM cognitive subscore (14–98). We calculated the gain in UK FIM+FAM between admission and discharge. There is no published Minimal Clinically Important Difference (MCID) for the UK FIM+FAM.^6^ Beninato *et al* identified a gain of 22 points or more on the total FIM scale as the MCID for patients with stroke.^15^ Using this cut-off, we divided the patients into two groups: those who achieved overall MCID and those who did not achieve MCID on discharge from the rehabilitation services. Using the same method, a gain of 17 or more on the motor FIM component and three or more points on the cognitive FIM components was taken as the MCID for the motor and cognitive domains, respectively.^15^

The RCS-E V.13 is a scale of complexity based on the medical, nursing and therapy resources required for the patient.^16^ In the current V.13, the total score ranges from 0 to 22, with 22 indicating the most complex needs. The NPDS is a 23-item, valid and reliable ordinal scale which assesses care and nursing needs during a stay in rehabilitation.^17^ Total scores range from 0 to 100, with higher scores indicating the need for higher levels of care. The NPCNA is a derived NPDS tool using an automated algorithm.^18^ It provides a timetable of care needs and a care package that would be required to meet those needs in the community. Using this information and the approximate cost of care per week (based on care agency costs in England), the algorithm estimates the total care hours per week.

The PCAT is a reliable ordinal scale assessing the complexity of rehabilitation needs across a variety of aspects from 1 to 3, with higher scores indicating increased care needs in that domain.^19^ It is used to determine a category (A–D) to assist in deciding which level of rehabilitation service is appropriate for those requiring specialist rehabilitation. According to the National Health Service England NHSE service specification, patients with category A and B needs require admission to Level 1 and Level 2 centres.^20^

Based on the discharge destination, we divided patients into two groups: those who could be discharged home and those who were discharged to residential nursing care.

### Data extraction

**Figure 1:**
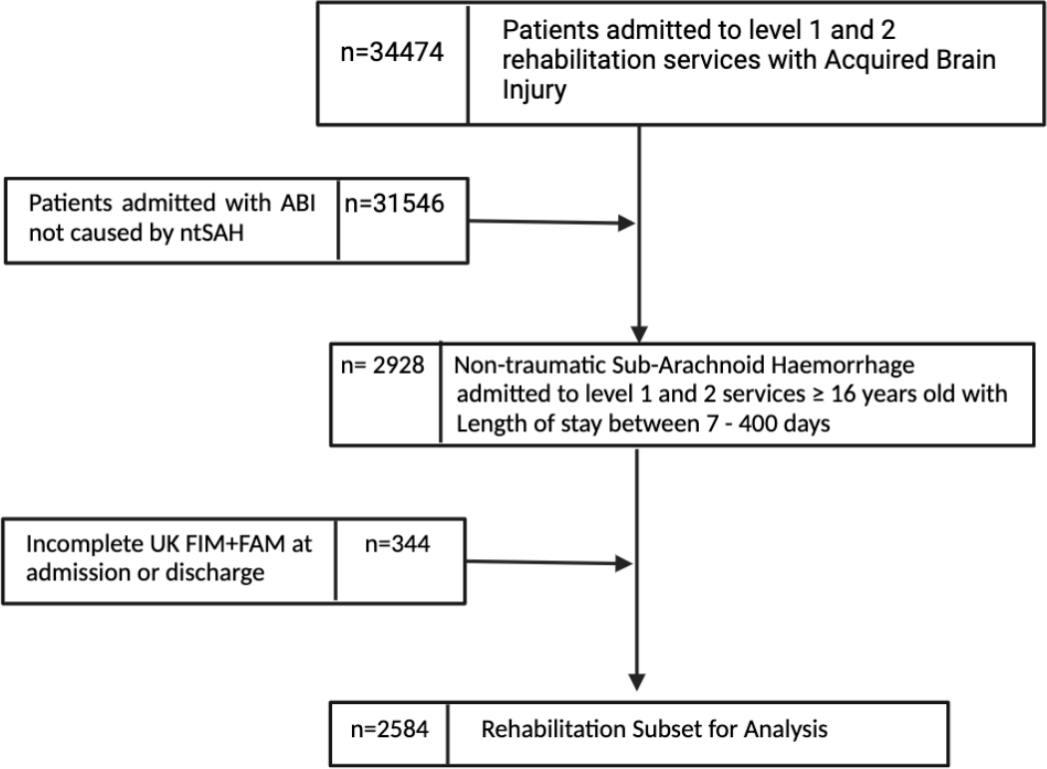
Data extraction process. ABI, Acquired Brain Injury, ntSAH, non-traumatic subarachnoid haemorrhage; UK FIM+FAM, UK Functional Independence Measure+Functional Assessment Measure.

The UKROC team extracted data for patients aged 16 years or older, admitted to a Level 1 or Level 2 specialist inpatient rehabilitation service, following ntSAH between March 2012 and March 2023. The study team included 2584 patients with complete UK FIM+FAM scores at both admission and discharge (see figure 1). The study team did not have direct access to the raw data that are available with UKROC.

### Statistics

All statistical analyses were performed in SPSS V.28. Summary statistics were used to represent the demographics of the population. To determine the functional gains made by patients, the difference in admission and discharge scores was analysed using paired-samples t-tests. For the comparison of length of stay, since the data were skewed, the non-parametric Mann-Whitney U test was used to compare the two groups. A targeted binary logistic regression model was used to identify factors which were associated with changes in the odds of achieving MCID on total FIM. We developed a large model including all variables available and then removed variables which were proving to have an insignificant effect (p values>0.150), except for certain key variables. The key variables were age, gender, ethnicity, length of stay, duration between referral to admission, NPDS and RCS-E V.13. The same process was followed to assess the odds of home discharge. Due to the nature of the regression model used in the assessment of our primary outcome and the others being exploratory variables, we have not performed any multiple testing correction.

### Patient public involvement

There was no patient and public involvement.

## Results

Table 1 shows the demographic information, nature and duration of rehabilitation services and discharge destination. The ethnicities were white British 74.1%, other white ethnicities 2.7%, British Asian/Asian 3.3%, black/black British 2.0% and other less common ethnicities 1.4%. Ethnicity data were missing for 16.5%. Almost two-thirds of patients were admitted to Level 2 services (65.2%) and one-third to Level 1 services (34.8%).

**Table 1:** Demographics, rehabilitation services and discharge destination

Parameters	Whole sample	
	Missing	n=2584
Age, mean (SD)	0	57.8 (13.2)
Patient gender, male:female (%)	1	41.1/58.8
Time since onset (days), median (IQR)	69	51.0 (31.0 to 87.0)
Stay (days), median (IQR)	0	69.0 (34.0 to 118.0)
Service parameters		
Admitted to Level 1/Level 2 services (%)	0	34.8/65.2
Days from referral to admission, median (IQR)	98	14 (6.0 to 31.0)
Discharge destination, n (%)	33	
Home		1616 (62.5)
Acute hospital		221 (8.6)
Nursing Home		256 (9.9)
Other inpatient rehabilitation		228 (8.8)
Residential care		102 (3.9)

### Functional changes

**Figure 2:**
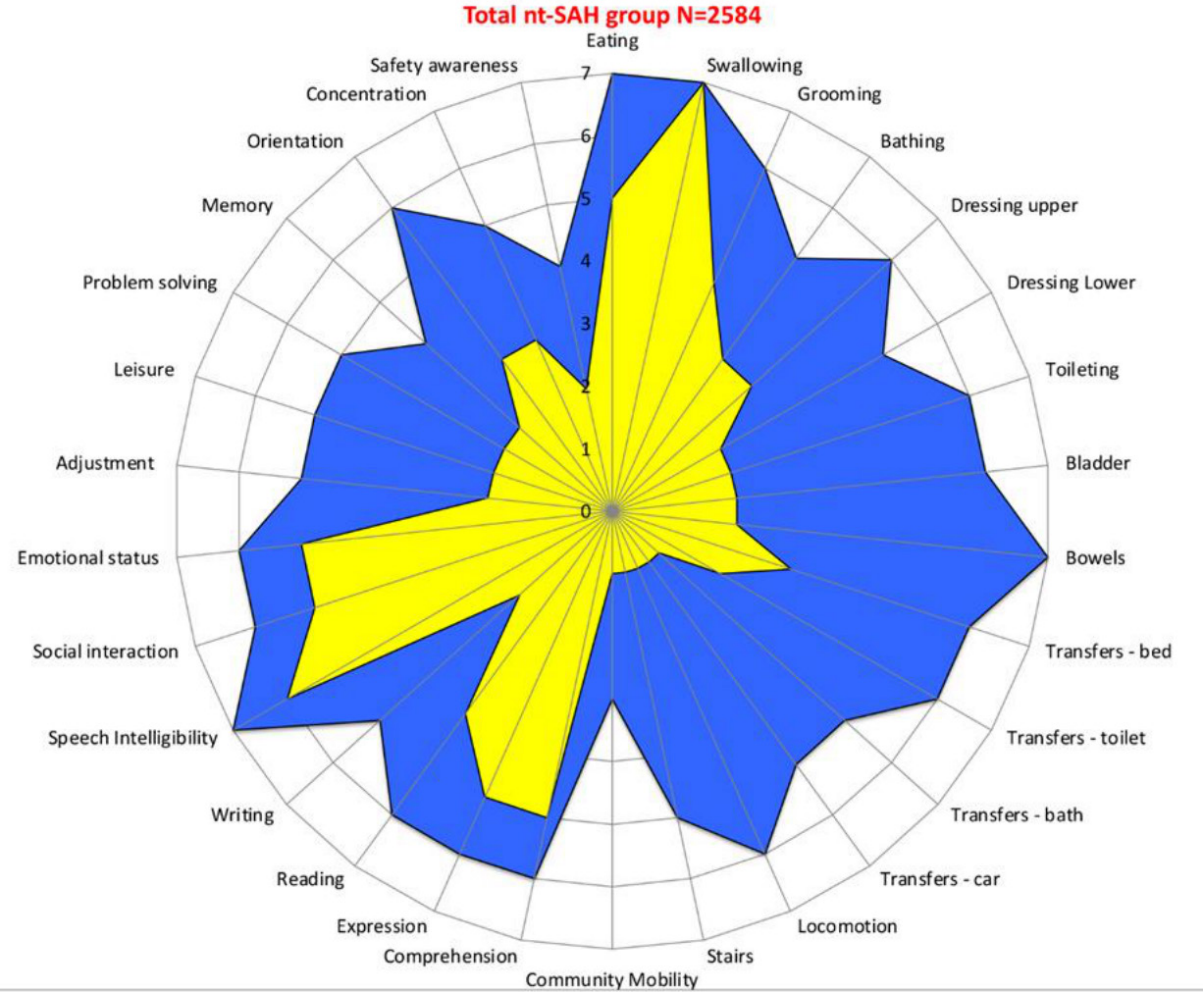
Summary of functional gains across different domains of UK FIM+FAM. Key: yellow=admission, blue=discharge. The radar chart (or ‘FAM splat’) provides a graphic representation of the disability profile from the FIM+FAM data. The 30-scale items are arranged as spokes of a wheel. Scoring levels from 1 (total dependence) to 7 (total independence) run from the centre outwards. Thus, a perfect score would be demonstrated as a large circle. This composite radar chart illustrates the median scores on admission and discharge for the ntSAH study population (n=2584). The yellow-shaded portion represents the median scores on admission for each item, while the blue-shaded area represents the median score on discharge. ntSAH, non-traumatic subarachnoid haemorrhage; UK FIM+FAM, UK Functional Independence Measure+Functional Assessment Measure.

Table 2 shows a summary of the UK FIM+FAM, NPCNA and NPDS scores on admission and discharge. The mean NPDS on admission was 34.1 (95% CI 33.3 to 34.9), which translates into mean care hours per week in the community of 75.9 (95% CI 74.1 to 77.7) with mean care costs per week £6306 (95% CI 6169 to 6442). There was an improvement in functional independence across all subsections of the UK FIM+FAM (figure 2). There was a mean increase in the total UK FIM+FAM score of 42.3 (95% CI 41.0 to 43.6, p value<0.001). The UK FIM+FAM efficiency (ie, mean FIM+FAM gain/length of stay for the service^21^) was 0.6.

**Table 2:** Summary of average admission scores, discharge scores and functional gains

	Admission mean (95% CIs)	Discharge mean (95% CIs)	Mean difference	(95% CIs)	t-test	P value (t-tailed)
Functional independence (UK FIM+FAM)						
Self-care	26.1 (25.6 to 26.7)	35.9 (35.4 to 36.5)	9.8	(9.5 to 10.2)	52.6	<0.001
Sphincter control	7.1 (6.9 to 7.3)	9.9 (9.7 to 10.0)	2.8	(2.6 to 2.9)	35.5	<0.001
Transfers	11.5 (11.2 to 11.9)	18.7 (18.4 to 19.1)	7.2	(6.9 to 7.5)	49.3	<0.001
Locomotion	6.5 (6.3 to 6.7)	11.7 (11.4 to 11.9)	5.2	(5.0 to 5.4)	50.1	<0.001
Communication	20 (19.7 to 20.4)	25.6 (25.3 to 26.0)	5.6	(5.4 to 5.8)	46.3	<0.001
Psychosocial	14.5 (14.2 to 14.7)	19.2 (18.9 to 19.5)	4.7	(4.5 to 4.9)	45.2	<0.001
Cognitive	14.7 (14.4 to 15.1)	21.7 (21.3 to 22.0)	6.9	(6.7 to 7.2)	50.9	<0.001
Subsection and total scores of FIM+FAM						
Motor subscore	51.1 (50.0 to 52.3)	76.2 (74.9 to 77.5)	25	(24.1 to 25.9)	56.3	<0.001
Cognitive subscore	49.2 (48.3 to 50.1)	66.5 (65.6 to 67.3)	17.3	(16.7 to 17.9)	57	<0.001
Total FIM+FAM	100.4 (98.4 to 102.3)	142.7 (140.5 to 144.8)	42.3	(41.0 to 43.6)	63.2	<0.001
Subsection scores and total scores of FIM						
Motor	42.2 (41.2 to 43.2)	62.7 (61.6 to 63.8)	20.5	(19.7 to 21.3)	53.4	<0.001
Cognitive	18.2 (17.8 to 18.5)	24 (23.6 to 24.3)	5.8	(5.6 to 6.0)	51.1	<0.001
Total FIM	60.3 (59.1 to 61.6)	86.6 (85.3 to 88.0)	26.3	(25.4 to 27.2)	57.9	<0.001
Dependency (NPDS/NPCNA)						
Total NPDS score	34.1 (33.3 to 34.9)	21.5 (20.6 to 22.7)	−12.7	(−13.3 to −12.0)	−40.2	<0.001
NPCNA care hours/week	75.9 (74.1 to 77.7)	49.2 (47.4 to 51.1)	−26.6	(−28.1, −25.2)	−36.7	<0.001
Care costs/week (£)	6305 (6169 to 6442)	4307 (4202 to 4154)	1998	(−2124 to −1872)	−31.1	<0.001

NPCNA, Northwick Park Care Needs Assessment; NPDS, Northwick Park Dependency Scale; UK FIM+FAM, UK Functional Independence Measure+Functional Assessment Measure.

The mean reduction in total NPDS score was 12.7 (95% CI 12.0 to 13.3, p value<0.001). According to the NPCNA algorithm, this results in a mean reduction in total care hours of 26.6 (95% CI 25.3 to 28.1, p value<0.001) and a mean saving in care costs of £1998 per week (95% CI 1872 to 2125, p value<0.001). This amounts to a saving of £103 584 per patient per year.

### Predictors of functional gains

**Figure 3:**
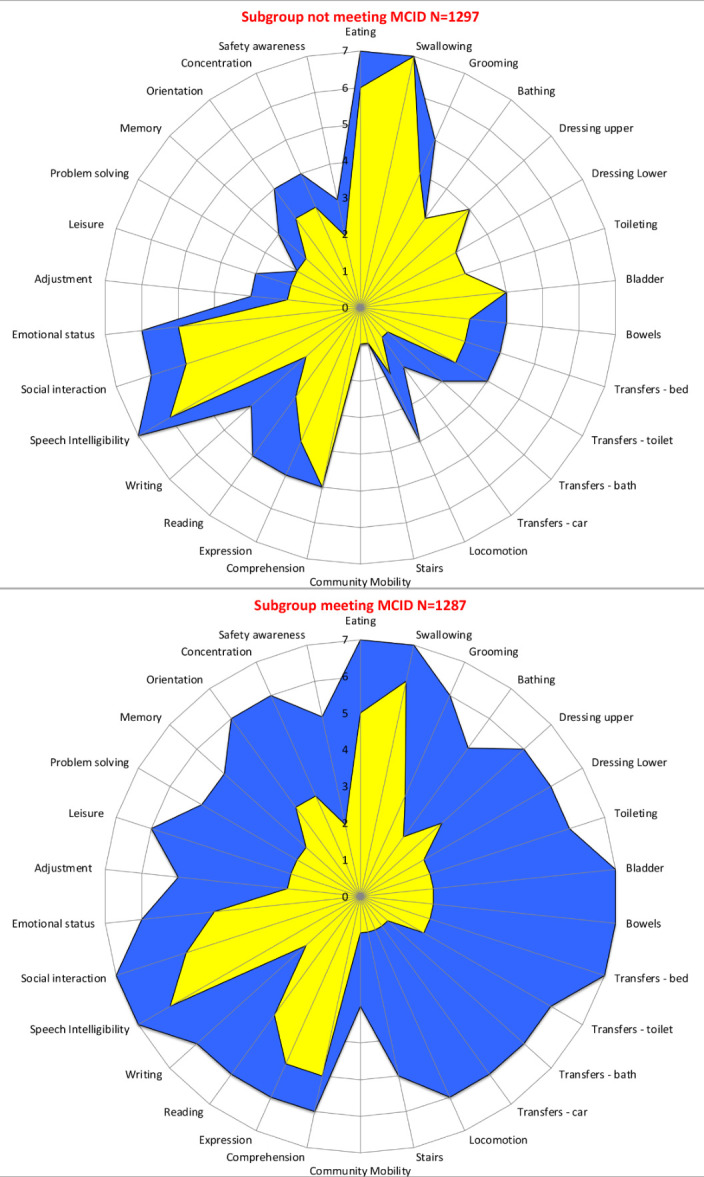
Summary of functional gains made across the MCID and non-MCID groups. Key: yellow=admission, blue=discharge. The radar chart (or ‘FAM splat’) provides a graphic representation of the disability profile from the FIM+FAM data. The 30 scale items are arranged as spokes of a wheel. Scoring levels from 1 (total dependence) to 7 (total independence) run from the centre outwards. Thus, a perfect score would be represented as a large circle. This composite radar chart illustrates the median scores on admission and discharge for the ntSAH study population (n=2584). The yellow-shaded portion represents the median scores on admission for each item, while the blue-shaded area represents the median score on discharge. FAM, Functional Assessment Measure; FIM, Functional Independence Measure; MCID, Minimal Clinically Important Difference; ntSAH, non-traumatic subarachnoid haemorrhage.

In total, 1287 (49.8%) made functional gains of 22 points or more in the total FIM score and reached MCID. In the motor domain, 1256 (48.6%) and in the cognitive domain, 1715 (66.4%) of patients achieved MCID. Figure 3 shows the functional gains across the different domains of the UK FIM+FAM. The patients in the group who did not meet the MCID were overall slightly more able on admission (figure 3). Even though they may not have met the MCID criteria, they still gained in different domains, particularly in transfers, speech, concentration, orientation, social interactions and emotional status. The areas where this group did not make gains were dressing, toileting, bathing, stairs and community mobility.

The factors on admission, influencing the odds of achieving the MCID are highlighted in table 3. Factors at admission associated with achieving MICD are (a) admission to a rehabilitation facility within 30 days of referral, (b) white British ethnicity, (c) low NPDS, (d) higher scores on locomotion, mobility and communication subsections of FIM/FAM and (e) higher PCAT scores on the vocational rehabilitation and behavioural sections. In comparison to white British individuals, people of Asian/British Asian ethnicity had significantly reduced odds of achieving MCID (0.360, p<0.001). Increased PCAT scores on admission, indicating more complex needs in the domains of specialist facilities and equipment (0.638, p≤0.001) and complex disability management (0.622, p≤0.001), were associated with decreased odds of achieving the MCID.

**Table 3:** Predictors of achieving the MCID in total FIM score

	Odds of overall MCID	95% CI		Significance
		Lower	Upper	
Admission age	0.996	0.987	1.005	0.369
Patient gender, female:male	1.185	0.956	1.470	0.122
Ethnicity*				
White (other)	0.560	0.287	1.095	0.090
Asian/British Asian	0.360	0.196	0.663	0.001
Black/black British	0.659	0.299	1.453	0.301
Other	1.016	0.416	2.479	0.972
Length of stay <90 days	0.798	0.628	1.015	0.066
Referral to admission <30 days	2.092	1.638	2.673	<0.001
NPDS total score	0.986	0.976	0.996	0.005
RCS-E V.13 total score	1.019	0.964	1.077	0.517
Admission UK FIM+FAM subsection scores				
Mobility (transfers)	0.946	0.918	0.975	<0.001
Locomotion	0.804	0.766	0.844	<0.001
Communication	1.045	1.028	1.062	<0.001
Cognitive	0.992	0.974	1.011	0.415
Admission PCAT scores				
Specialist facilities and equipment	0.638	0.534	0.762	<0.001
Vocational rehabilitation needs	1.487	1.299	1.704	<0.001
Family support	1.210	0.943	1.552	0.133
Complex disability management	0.622	0.473	0.819	<0.001
Behavioural management	1.486	1.223	1.806	<0.001

* White British ethnicity is the comparator level.

MCID, Minimal Clinically Important Difference; NPDS, Northwick Park Dependency Scale; PCAT, Patient Categorisation Tool; RCS-E, Rehabilitation Complexity Scale; UK FIM+FAM, UK Functional Independence Measure+Functional Assessment Measure.

### Predictors of home discharge

The predictors of home discharge are given in table 4. The length of stay of less than 90 days (1.457, p=0.007) was associated with higher odds of home discharge. Achieving both motor MCID (2.733, p≤0.001) and cognitive MCID (2.345, p≤0.001) was independently associated with increased odds of home discharge. In addition, higher PCAT scores on medicolegal issues (0.689, p≤0.001), expected duration of stay in rehabilitation (0.763, p=0.019) and tracheostomy/ventilation (0.644, p=0.011) were associated with reduced odds of home discharge.

**Table 4:** Predictors of home discharge

	Odds of home discharge	95% CI		Significance
		Lower	Upper	
Admission age	1.003	0.993	1.013	0.520
Patient gender, female:male	1.223	0.952	1.570	0.116
Ethnicity*				
White (other)	0.734	0.529	1.017	0.063
Asian/British Asian	0.479	0.210	1.093	0.080
Black/black British	0.608	0.290	1.274	0.187
Other	0.719	0.290	1.785	0.477
Length of stay <90 days	1.457	1.111	1.910	0.007
Referral to admission <30 days	0.889	0.670	1.180	0.416
NPDS total score	0.988	0.977	0.999	0.040
RCS-E V.13 total score	0.988	0.928	1.053	0.720
FIM change scores				
Gain of score≥22	1.078	0.591	1.967	0.806
Gain of cognitive subscore≥3	2.345	1.730	3.180	<0.001
Gain of motor subscore≥17	2.733	1.527	4.893	<0.001
Admission PCAT scores				
Tracheostomy/ventilation	0.644	0.459	0.905	0.011
Medicolegal issues	0.689	0.585	0.812	<0.001
Expected duration of stay	0.763	0.609	0.956	0.019

* White British ethnicity is the comparator level.

FIM, Functional Independence Measure; NPDS, Northwick Park Dependency Scale; PCAT, Patient Categorisation Tool; RCS-E, Rehabilitation Complexity Scale.

## Discussion

This is the first nationwide study of outcomes following specialist rehabilitation following ntSAH in England. The study had a large representative population of 2584 patients undergoing specialist rehabilitation (88.3%) and is the largest reported study on rehabilitation outcomes following ntSAH in the world. The demographics of our cohort are like that of epidemiological studies on ntSAH.^22 23^ The average length of stay in this study was longer than in many studies (69 days compared with 42–49 days), which is due to the complex needs and highly specialised nature of the rehabilitation provided to patients in Level 1 and Level 2 facilities in England. Other series included people with less complex rehabilitation needs, the equivalent of UK Level 3 rehabilitation programmes.^21 23 24^

Our cohort showed a mean improvement of 42.3 points for the UK FIM+FAM and 26.3 points on the UK FIM. These functional gains are like those seen in multiple smaller studies on rehabilitation gains after ntSAH.^2124^,^26^ Our results further validate the beneficial effect of rehabilitation on functional outcomes for survivors of ntSAH. Functional gains were seen in all domains of the UK FIM+FAM, which shows the widespread benefits of rehabilitation (figure 2). These were reflected in a reduction in their ongoing care needs in the community, resulting in substantial savings in care costs averaging over £100 000 per patient per year based on the UKROC algorithm.

The ethnicities of the patients play a significant role in outcomes following rehabilitation. A study from the USA showed that, among patients who underwent rehabilitation following traumatic brain injury, those of non-Hispanic black ethnicity had lower motor, cognitive and total FIM scores at discharge.^27 28^ In our study, people of Asian ethnicity had lower odds of achieving MCID compared with white British patients. Ours is the first study to explore the impact of ethnicity on outcomes following rehabilitation for ntSAH in the UK. Like the studies in the US on traumatic brain injury and stroke, our results also showed that ethnic differences do have an impact on rehabilitation outcomes following ntSAH. These findings suggest a need for culturally sensitive rehabilitation programmes aimed at patients from ethnic minority groups.

Following a stroke or traumatic brain injury, patients who experienced delays of more than 30 days for admission to a rehabilitation ward had poorer outcomes.^29^,^31^ In our cohort of ntSAH, those who waited for more than 30 days after referral for transfer to the rehabilitation ward had a significantly lower chance of achieving MCID. This could indicate that delays in transfer to a specialist rehabilitation unit impact negatively on outcome following ntSAH. However, the association is not necessarily causative, as the increased waiting time could simply be an indicator of the severity of the initial presentation of ntSAH. Our results highlight the importance of developing rehabilitation services alongside acute care to start rehabilitation as early as possible after ntSAH.

As expected, patients with higher requirements for specialist equipment and facilities, and for ongoing complex disability management, had reduced odds of achieving the MCID across all FIM+FAM domains; however, there was no significance in relation to home discharge. In our model, the gain of MICD in total FIM did not show significance in relation to home discharge. This may be explained within the context of the statistical model used, as it simultaneously accounted for both motor and cognitive MCID. The patients achieving overall MCID will already be accounted for in the two subsections.

Patients who had high medical needs, as highlighted by the PCAT score on medical/surgical acuity and tracheostomy/ventilation scores, were less likely to achieve MCID. This highlights the significant impact of medical complications on rehabilitation outcomes and the need for hyperacute rehabilitation units that can manage these patients with complex needs within the context of rehabilitation.^32^ Patients with identified needs for vocational rehabilitation on the PCAT were more likely to achieve MCID across all FIM+FAM domains. The PCAT is a measure of the complexity of rehabilitation needs on admission. This association is because vocational rehabilitation is only likely to be relevant for those who are making rapid functional gains towards independence.

Around a third of patients in this cohort needed residential care. Age, gender and ethnicity did not affect discharge destination. Patients who achieved MCID in either the motor or cognitive domains were more than two times as likely to be discharged home. This highlights the benefits of specialist rehabilitation for complex needs and the value of targeted plans for specific areas of deficit. Small benefits can make a substantial difference, especially in the cognitive domains.

### Strengths

The study has the largest cohort size among the studies on outcomes following the rehabilitation of patients with ntSAH. As we used data from UKROC collected during usual clinical practice, the results are generalisable to all Level 1 and Level 2 rehabilitation services throughout England. In this study, we had to exclude only a small proportion of the original population from the analysis population due to incomplete data. This is a particular strength compared with studies of a similar nature and points to the robust nature of the data collection process for UKROC.

### Limitations

The UKROC database categorised all patients with ‘non-traumatic subarachnoid haemorrhage’ together into a single category; therefore, we could not investigate the impact of the aetiology of ntSAH on rehabilitation outcomes. The most frequent cause of ntSAH is aneurysmal bleed. As this is a large, non-selective population database, we assume that the majority of patients in our cohort had an aneurysmal bleed. The UKROC database also does not have any data on the acute stages of each patient, like the severity of the ntSAH on admission, rebleed and vasospasm. There are major limitations in using the MCID based on the FIM as a measure of good outcomes, as the goals of rehabilitation may be different for different patients, and the FIM is known to have limited sensitivity in patients with highly complex needs following acquired brain injury.^12^ Data with a more goal-orientated outcome measure, like the Goal Assessment Scale, could have provided more information on patient-centred outcomes. Although the UKROC database allows for goal attainment to be entered, few centres complete these data, as NHS England does not mandate them.

## Summary

### Conclusions

This study is the first on the overall outcomes for patients with ntSAH in England. It has shown that rehabilitation is an effective method in improving functional outcomes across all total FIM+FAM, motor FIM+FAM and cognitive FIM+FAM. Around 50% of patients admitted to Level 1 and Level 2 rehabilitation facilities achieved the MCID on FIM scores. The predictors of achieving MCID include ethnicity, time from referral to admission, admission NPDS and admission functionality scores. The predictors for home discharge include length of stay, admission functional dependency scores and achievement of the MCID in motor and/or cognitive domains of the FIM.

## Data Availability

Data are available upon reasonable request.

## References

[1] Sundström J, Söderholm M, Söderberg S (2019). Risk factors for subarachnoid haemorrhage: a nationwide cohort of 950 000 adults. Int J Epidemiol.

[2] Claassen J, Park S (2022). Spontaneous subarachnoid haemorrhage. Lancet.

[3] Krishnamurthi RV, Ikeda T, Feigin VL (2020). Global, Regional and Country-Specific Burden of Ischaemic Stroke, Intracerebral Haemorrhage and Subarachnoid Haemorrhage: A Systematic Analysis of the Global Burden of Disease Study 2017. Neuroepidemiology.

[4] Roquer J, Cuadrado-Godia E, Guimaraens L (2020). Short- and long-term outcome of patients with aneurysmal subarachnoid hemorrhage. Neurology (ECronicon).

[5] Taufique Z, May T, Meyers E (2016). Predictors of Poor Quality of Life 1 Year After Subarachnoid Hemorrhage. Neurosurgery.

[6] Bederson JB, Connolly ES, Batjer HH (2009). Guidelines for the Management of Aneurysmal Subarachnoid Hemorrhage. Stroke.

[7] Galea JP, Dulhanty L, Patel HC (2017). Predictors of Outcome in Aneurysmal Subarachnoid Hemorrhage Patients. Stroke.

[8] van Gijn J, Kerr RS, Rinkel GJE (2007). Subarachnoid haemorrhage. Lancet.

[9] Rivero-Arias O, Gray A, Wolstenholme J (2010). Burden of disease and costs of aneurysmal subarachnoid haemorrhage (aSAH) in the United Kingdom. Cost Eff Resour Alloc.

[10] Nayar M, Vanderstay R, Siegert RJ (2016). The UK Functional Assessment Measure (UK FIM+FAM): Psychometric Evaluation in Patients Undergoing Specialist Rehabilitation following a Stroke from the National UK Clinical Dataset. PLoS One.

[11] Turner-Stokes L, Lafeuillee G, Francis R (2022). Functional outcomes and cost-efficiency of specialist in-patient rehabilitation following spinal cord injury: a multi-centre national cohort analysis from the UK Rehabilitation Outcomes Collaborative (UKROC). Disabil Rehabil.

[12] Turner-Stokes L, Williams H, Bill A (2016). Cost-efficiency of specialist inpatient rehabilitation for working-aged adults with complex neurological disabilities: a multicentre cohort analysis of a national clinical data set. BMJ Open.

[13] UK Rehabilitation Outcomes Collaboration (2024). Clinical tools.

[14] Turner-Stokes L, Nyein K, Turner-Stokes T (1999). The UK FIM+FAM: development and evaluation. *Clin Rehabil*.

[15] Beninato M, Gill-Body KM, Salles S (2006). Determination of the minimal clinically important difference in the FIM instrument in patients with stroke. Arch Phys Med Rehabil.

[16] Turner-Stokes L, Williams H, Siegert RJ (2010). The Rehabilitation Complexity Scale version 2: a clinimetric evaluation in patients with severe complex neurodisability. *J Neurol Neurosurg Psychiatry*.

[17] Siegert R, Turner-Stokes L (2010). Psychometric evaluation of the Northwick Park Dependency Scale. *J Rehabil Med*.

[18] Turner-Stokes L, Nyein K, Halliwell D (1999). The Northwick Park Care Needs Assessment (NPCNA): a directly costable outcome measure in rehabilitation. Clin Rehabil.

[19] Turner-Stokes L, Krägeloh CU, Siegert RJ (2019). The patient categorisation tool: psychometric evaluation of a tool to measure complexity of needs for rehabilitation in a large multicentre dataset from the United Kingdom. Disabil Rehabil.

[20] NHS England (2013). *NHS standard contract for specialised rehabilitation for patients with highly complex needs (all ages*).

[21] Dombovy ML, Drew-Cates J, Serdans R (1998). Recovery and rehabilitation following subarachnoid haemorrhage. Part I: Outcome after inpatient rehabilitation. Brain Inj.

[22] Al-Khindi T, Macdonald RL, Schweizer TA (2010). Cognitive and functional outcome after aneurysmal subarachnoid hemorrhage. Stroke.

[23] Lindner A, Brunelli L, Rass V (2023). Long-Term Clinical Trajectory of Patients with Subarachnoid Hemorrhage: Linking Acute Care and Neurorehabilitation. Neurocrit Care.

[24] O’Dell MW, Watanabe TK, De Roos ST (2002). Functional outcome after inpatient rehabilitation in persons with subarachnoid hemorrhage. Arch Phys Med Rehabil.

[25] Chua KSG, Loke JJY, Lim CJ (2024). Rehabilitation outcome after acute subarachnoid haemorrhage: the role of early functional predictors and complications. Singapore Med J.

[26] Stabel HH, Pedersen AR, Johnsen SP (2017). Rupture of a non-traumatic anterior communicating artery aneurysm: Does location of aneurysm associate with functional independence following post-acute in-patient neurorehabilitation?. Top Stroke Rehabil.

[27] Garcia JJ, Warren KL (2019). Race/Ethnicity Matters: Differences in Poststroke Inpatient Rehabilitation Outcomes. Ethn Dis.

[28] Schupper AJ, Hardigan TA, Mehta A (2023). Sex and Racial Disparity in Outcome of Aneurysmal Subarachnoid Hemorrhage in the United States: A 20-Year Analysis. Stroke.

[29] Sirois MJ, Lavoie A, Dionne CE (2004). Impact of transfer delays to rehabilitation in patients with severe trauma. Arch Phys Med Rehabil.

[30] Salter K, Jutai J, Hartley M (2006). Impact of early vs delayed admission to rehabilitation on functional outcomes in persons with stroke. J Rehabil Med.

[31] Bae SW, Lee M-Y (2023). Association Between Initiation of Rehabilitation and Length of Hospital Stay for Workers with Moderate to Severe Work-Related Traumatic Brain Injury. Saf Health Work.

[32] Turner-Stokes L, Bavikatte G, Williams H (2016). Cost-efficiency of specialist hyperacute in-patient rehabilitation services for medically unstable patients with complex rehabilitation needs: a prospective cohort analysis. BMJ Open.

